# Economy benefits of running in advanced footwear technology shoes remain with plantarflexion fatigue

**DOI:** 10.1007/s00421-026-06190-0

**Published:** 2026-03-14

**Authors:** Benjamin Bidois, Christiaan Cumming, Marlene Giandolini, Anh Phong Nguyen, Kim Hébert-Losier

**Affiliations:** 1 Te Wānanga Waiora Division of Health, University of Waikato, Tauranga, New Zealand; 2 School of Science and Technology, University of New England, Armidale, Australia; 3 Research and Sport Sciences, Annecy, France; 4 Neuromusculoskeletal lab Bruxelles, Université Catholique de Louvain, Institut de Recherche Expérimentale et Clinique, Brussels, Belgium; 5 Research & Development, The Running Clinic TM, Lac-Beauport, Canada

**Keywords:** Calf muscle, Running performance, Super shoes, Fatigue

## Abstract

**Purpose:**

We investigated the effects of plantarflexion fatigue on running economy (RE) response to advanced footwear technology (AFT) in a group of heterogenous runners. Secondary aims were to examine the relationships between plantarflexion power and RE responses to AFT, and effects of plantarflexion fatigue and footwear on biomechanical measures.

**Methods:**

Sixty-four runners completed two laboratory sessions. Session one involved a peak oxygen uptake test. Session two included two RE tests in both a Control and AFT shoe before plantarflexion fatigue, and two rounds of plantarflexion fatigue before reassessment of RE in one of the two shoes (counterbalanced). Plantarflexion power was assessed at baseline, and before and after each fatigue protocol. Video-based running biomechanics were recorded during all RE tests.

**Results:**

RE measures were improved in AFT (35.3 ± 5.0 mL/kg/min, 12.5 ± 1.8 W/kg, 4.18 ± 0.40 J/kg/m) versus Control (36.8 ± 5.2 mL/kg/min, 13.1 ± 1.9 W/kg, 4.37 ± 0.40 J/kg/m), and better pre-fatigue (35.7 ± 5.1 mL/kg/min, 12.7 ± 1.8 W/kg, 4.25 ± 0.40 J/kg/m) than post-fatigue (36.4 ± 5.2 mL/kg/min, 12.8 ± 1.9 W/kg, 4.30 ± 0.42 J/kg/m). Plantarflexion power decreased 6.5% post-fatigue. Despite these significant effects of AFT and plantarflexion fatigue on RE, no interactions were observed (*P* ≥ 0.476). Baseline plantarflexion power and changes in power did not correlate with AFT responses (*P* ≥ 0.566). Statistically significant differences indicated AFT reduced ground contact time and foot strike angle and increased cycle time and duty factor, with plantarflexion fatigue increasing cycle time and duty factor; however, estimates of differences were generally within the limits of instrumental resolution.

**Conclusion:**

Plantarflexion fatigue and plantarflexion power were not associated with RE responses to AFT, opposing the idea that plantarflexion strength explains variability in AFT response.

Trial registration: Australian New Zealand Trials Registry, ACTRN12624000753550, 18th June 2024.

## Introduction

The triceps surae are the main plantarflexors and its muscles all share a common distal Achilles tendon insertion (Maffulli 1999; Spina 2007). Greater plantarflexion strength (Arampatzis et al. 2006; Bohm et al. 2021) and Achilles tendon stiffness (Nguyen et al. 2025) have been associated with superior running economy (RE), with RE positively linked to running performance (Hoogkamer et al. 2016). The contribution of the triceps surae to the total metabolic cost of running has been estimated at approximately 25% in highly trained individuals and up to 40% in untrained individuals (Fletcher and MacIntosh 2015). While training may enhance the efficiency of the triceps surae, fatigue is also likely to alter the contribution of the plantarflexors during running (Saldanha et al. 2008).

Evidence suggests advancements in running shoe technology improve RE measures by 4.0% on average across recreational and elite runners (Hébert-Losier et al. 2022a; Hoogkamer et al. 2018; Hunter et al. 2019; Joubert et al. 2024; Knopp et al. 2023; Schwalm et al. 2025; Werkhausen et al. 2024), with accompanying performance benefits seen across running distances (Bermon et al. 2021; Muniz-Pardos et al. 2021). For instance, all world records from the 5 km to the marathon have been broken by runners wearing advanced footwear technology (AFT) (Muniz-Pardos et al. 2021), which typically have more cushioning and a stiff element within the midsole. The midsole materials of AFT (e.g., polyether block amide) is more responsive and compliant than traditional foam, while remaining lightweight regardless of the additional elements (Burns and Tam 2020; Hébert-Losier and Pamment 2023).

Despite the reported RE and performance benefits of AFT, considerable interindividual variability is apparent. For example, changes in oxygen consumption measured in both world class and amateur male runners wearing AFT ranged from + 11.4% (benefit) to -11.3% (detriment) and + 9.7% to -1.1% compared to traditional lightweight racing shoes, respectively (Knopp et al. 2023). Performance variability has also been reported when wearing AFT. Hébert-Losier et al. (2022a) reported 61% of male recreational runners ran their fastest 3 km treadmill time trials when wearing an AFT shoe, whereas the remaining 39% of runners recorded their fastest times wearing traditional road racing flats (22%) or their own shoes (17%). Placebo effects have been proposed to explain some of the variability in RE when wearing AFT (Hébert-Losier et al. 2022a; Hoogkamer et al. 2019; Hunter et al. 2019), although Hébert-Losier et al. (2025) found no significant placebo effect on RE measures. Other factors that may underpin AFT variability include running speeds (i.e., faster running speeds are linked with greater benefits) (Day and Hahn 2020; Joubert et al. 2023; McLeod et al. 2020; Paradisis et al. 2023) and plantarflexion strength, i.e., lack of plantarflexion strength is thought to limit the RE benefits of shoes containing a stiff element (Madden et al. 2016; Ortega et al. 2021). If plantarflexion strength is indeed a factor that contributes to the variability in AFT responses, then RE benefits linked to wearing AFT are likely to diminish as the triceps surae muscles fatigue during a long-distance run, especially given recent findings that carbon-plated shoes induce greater plantarflexion fatigue during half-marathon treadmill running (Perrin et al. 2025). Although maximal voluntary isometric contractions are commonly used in assessment of plantarflexion function and fatigue (Avela et al. 1999; Finni et al. 2003; Murray et al. 2019; Perrin et al. 2025; Petersen et al. 2007; Play et al. 2024; Saldanha et al. 2008), the use of plantarflexion power (Hébert-Losier et al. 2023) is a more dynamic and running-specific test of plantarflexion function (Komi 2000) deemed relevant in the context of running in AFT.

Therefore, we aimed to investigate the effects of plantarflexion fatigue on RE responses to AFT shoes in a heterogeneous cohort of runners. We hypothesised that the RE benefits linked with wearing AFT would be superior before compared to after a plantarflexion fatigue protocol. Our secondary aims were to investigate the relationship between baseline plantarflexion power and RE responses to AFT, the relationship between changes in plantarflexion power and changes in RE from pre-to-post plantarflexion fatigue, as well as the effects of plantarflexion fatigue and footwear on video-based running biomechanic measures.

## Methods

### Ethics approval

The Human Research Ethics Committee granted ethical approval [HREC(HECS)2024#11] before participant recruitment. We followed the University of Waikato Human Research Ethics Regulations, the Declaration of Helsinki, New Zealand’s Health Research Guidelines (Health Research Council, 2007), and UNICEF’s guide for research involving children (Graham et al. 2013). Participants provided informed consent with approval also sought from legal guardians of participants younger than 16 years of age. The trial was preregistered in the Australian New Zealand Trials Registry (ACTRN12624000753550).

### Participants

**Fig. 1:**
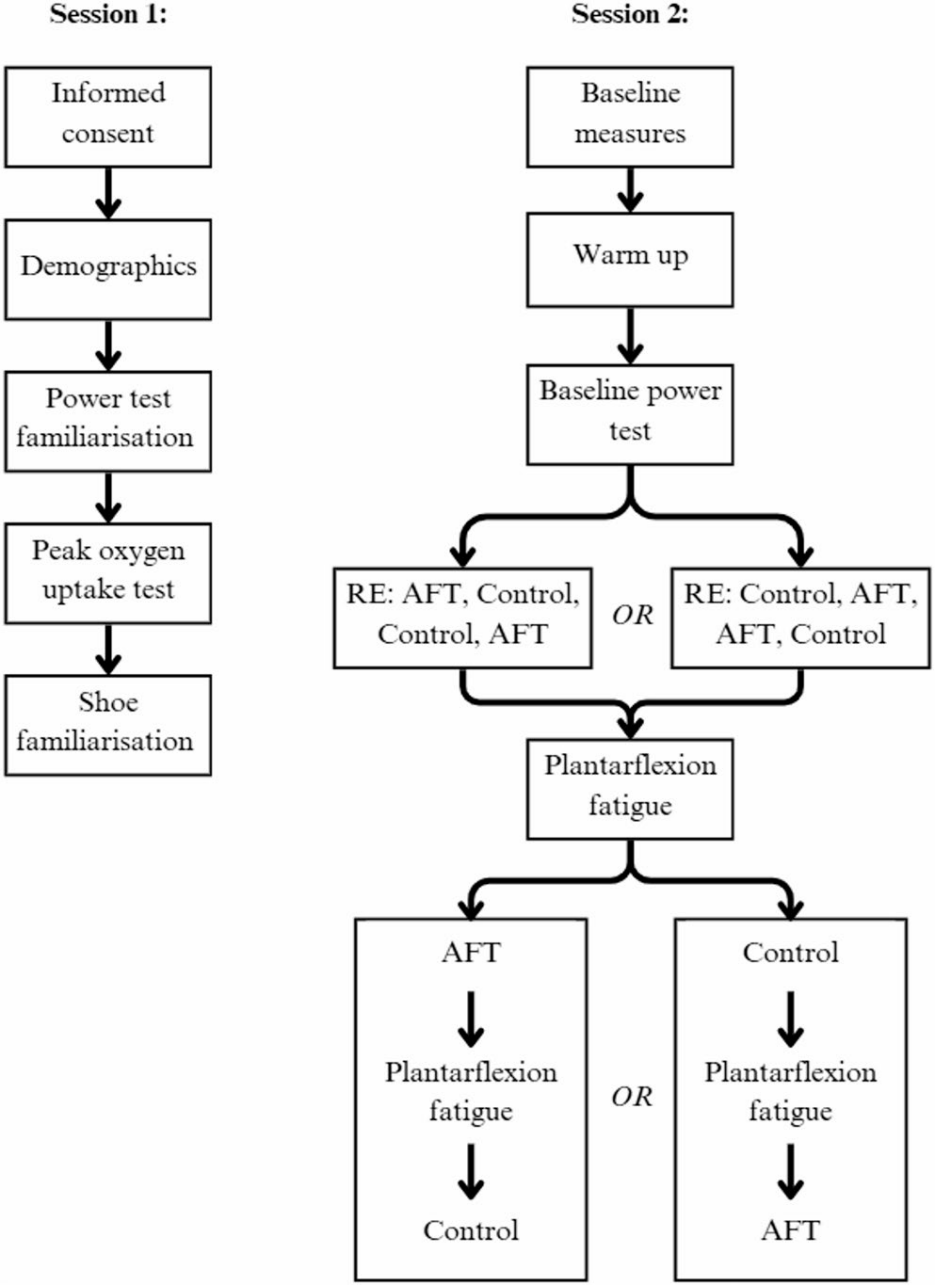
Experimental flow diagram. AFT, Salomon Phantasm S/Lab 2; Control, Salomon Aero Glide 2; RE, running economy

Sixty-four participants completed this study as part of a larger project that had a targeted sample size of 66 participants based on a priori sample size calculations (G*Power, 3.1.9.7) (i.e., detect *large* effect size differences between three functionally distinct groups, with *f* = 0.4, *alpha* = 0.05, *beta* = 0.20). Ultimately, 64 participants completed the study, indicating an ability to detect a *moderate* (*d* = 0.36) two-tailed effect size difference between paired means. All participants could run for at least 30 min, had been running at least once a week for six months, and fitted the available shoe sizes (Men US5 to US13). Participants were excluded if they reported having an injury within the month prior to participation (Yamato et al. 2015). The broad inclusion criteria of participants were to increase the generalisation of results. Participants were recruited through word-of-mouth, posters, running clubs, and social media advertisement, and tested within a 4-month period. All participants attended two laboratory sessions (Fig. 1) of 90 to 120 min in duration (5.3 ± 4.6 days apart) and did not exercise on the day of testing sessions.

### Session one

Session one was dedicated to informed consent, baseline demographics, familiarisation to test procedures, peak oxygen uptake test, and shoe familiarisation (Fig. 1). Specially, session one collected demographic information regarding their sex, age, mass, height, years of regular running (running once per week), 5 km personal best time in the last 6 months, and running classification (i.e., category and level) (McKay et al. 2022). Participants were then familiarised with the eccentric-concentric plantarflexion power test used in session two, following similar procedures to those reported elsewhere (Hébert-Losier et al. 2022b; Silbernagel et al. 2006). Participants stood on the edge of a flat steel step within a squat cage, where they completed three bodyweight repetitions of the eccentric-concentric plantarflexion movement as familiarisation and warm-up, followed by three repetitions with a weighted vest set to 30% of the participants’ mass after two minutes of rest. Participants were instructed to perform the test as strongly and quickly as possible. Subsequently, a peak oxygen uptake test was conducted running on a treadmill (h/p/cosmos^®^ Pulsar 3p, Germany) set to 0% incline, with running speeds increasing 0.5 km/h every minute until volitional exhaustion (Van Hooren and Lepers 2023). Self-reported best 10 km time defined participants’ starting velocities: 8 km/h (≥ 50 min), 10 km/h (40 to 49 min), 12 km/h (35 to 39 min), or 14 km/h (< 35 min). An average oxygen consumption was recoded every 15 s using a True One 2400 metabolic cart (Parvo Medics, Salt Lake City, UT) calibrated using a 3 L syringe and a gas mixture of 4.003% carbon dioxide and 16.00% oxygen. Peak oxygen uptake was defined by the average of two adjacent 15-second oxygen consumption values (mL/kg/min) which produces the highest number. At the conclusion of the test, the running velocity of the last fully completed stage was recorded and used to set subsequent RE trials to 70% of velocity at peak oxygen uptake (*v*VO_2peak_). After five minutes of recovery, participants ran at a self-selected speed for two minutes in both the Control (Salomon Aero Glide 2) and AFT (Salomon S/Lab Phantasm 2) shoe used for experimentation (Table 1). The shoe conditions were selected so the main distinguishing feature was the absence (Control) or presence (AFT) of a carbon fibre plate and polyether block amide foam (i.e., AFT shoe technology).

**Table 1:**
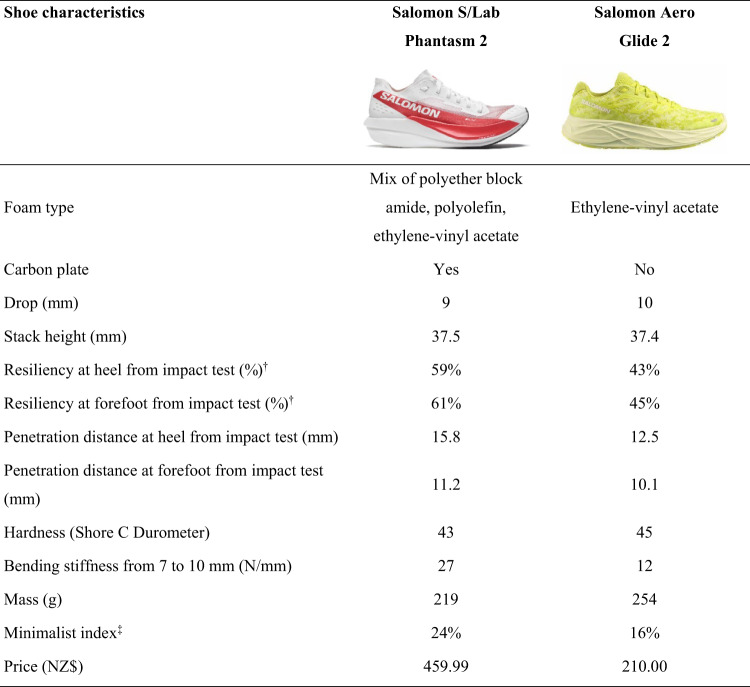
Experimental shoe characteristics

Data for a Men US9 shoe size

^†^Resiliency (ratio of drop height to rebound height) reflects the energy return based on dropping a 5 kg mass from a height of 23 cm onto the shoe and measuring the rebound height

^‡^Minimalist index range: 0% (lowest) to 100% (highest) degree of minimalism (Esculier et al. 2015)

### Session two

Session two was dedicated to a within-session assessment of RE in both experimental shoes, before and after plantarflexion fatigue. In this session, participants completed a total of four RE trials before plantarflexion fatigue and two rounds of plantarflexion fatigue, each round immediately followed by one RE trial (Fig. 1).

Before plantarflexion fatigue, baseline plantarflexion power and pre-fatigue RE measures were collected. Participants completed four six-minute RE tests at 70% of *v*VO_2peak_, each separated by five minutes of seated rest. Tests were completed in either the Control or AFT shoe using a mirrored crossover design (i.e., AFT-Control-Control-AFT or Control-AFT-AFT-Control). This approach helps minimise the risk of order effects on RE measures (Bradley 1958). Two RE tests in each shoe condition is recommended to reduce intra-individual variability (Barrons et al. 2024). Every 15 s during the RE tests, averages of oxygen consumption, volume of carbon dioxide, and respiratory exchange ratio (RER) were recorded. A 30-second sagittal plane slow motion video (240 fps, 8-bit) was recorded at the fifth minute of each RE test using an iPad (Pro, iOS 18.5) positioned 210 cm perpendicular from the treadmill’s centre on the left-side of participants and 30 cm off the ground. Given the sampling frequency, resolution errors for time-related variables are 4.2 ms. Additionally, blood lactate concentration samples (Lactate-Pro 2 analyser, Arkray inc., Kyoto, Japan) were collected at the end of each RE test.

**Fig. 2:**
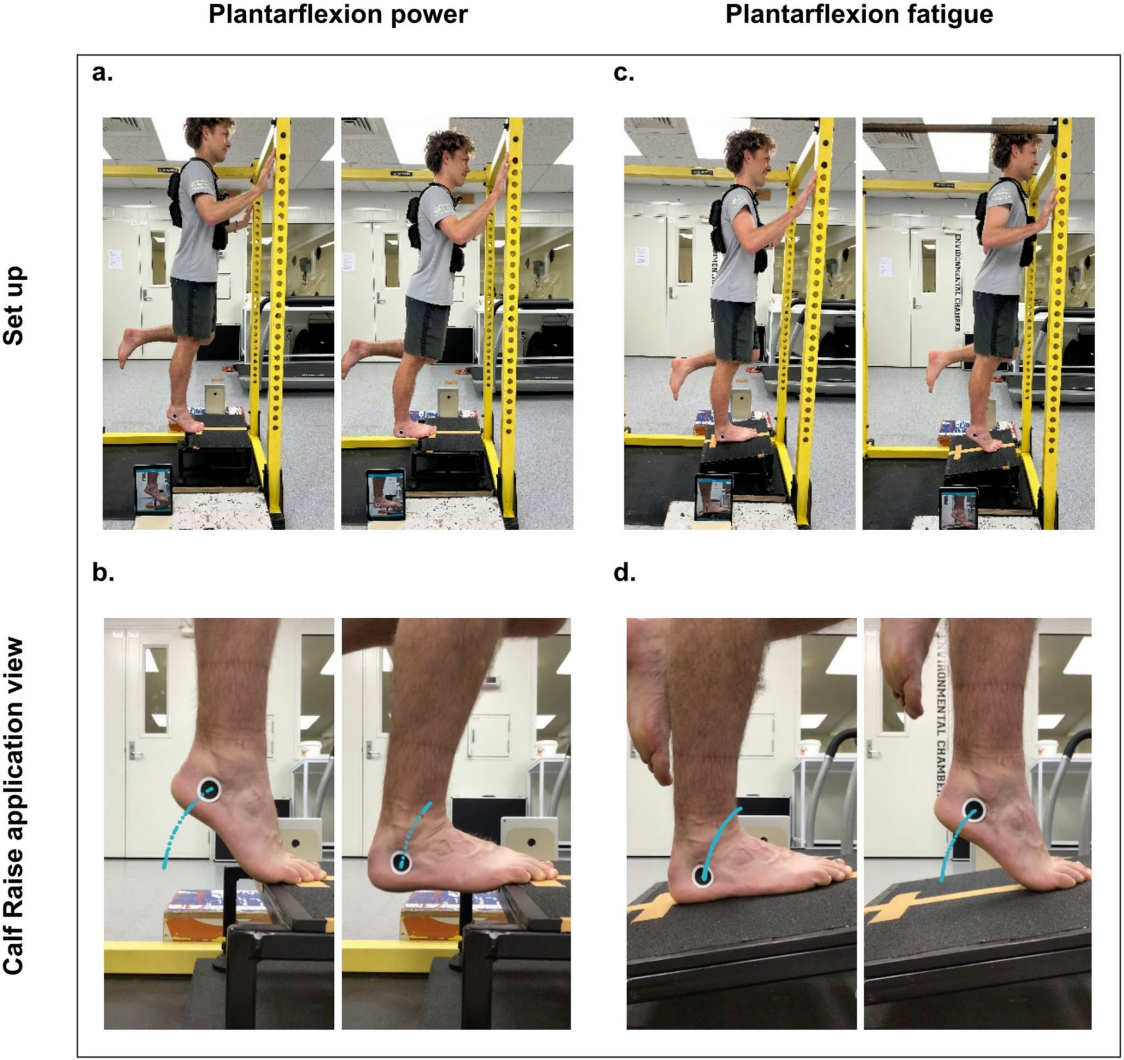
Illustrations of plantarflexion power and fatigue set up and Calf Raise application processing. (**a**, **b**) Plantarflexion power test is performed on a flat steel platform with 30% added body weight, where participants begin at the top of the raise (heel as high as possible) and move their heel down (eccentric) and up (concentric) as fast and strong as possible. (**c**, **d**) Plantarflexion fatigue is performed on a 10° inclined steel platform once with body weight only and once with 30% added body weight, where participants perform as many single-leg calf raise repetitions as possible, moving their heel up (concentric) and down (eccentric) to the beat of a metronome

Before plantarflexion fatigue, participants completed a pre-fatigue plantarflexion power test using their right leg. To develop plantarflexion fatigue, participants completed one set of single-leg body-weighted calf raises to failure (Hébert-Losier et al. 2022b) on a 10° inclined steel step, starting with the left leg and then the right leg. Participants immediately completed another set of single-leg calf raises while wearing the 30% body-weighted vest. Figure 2 illustrates the set up for the plantarflexion power test and fatigue protocol. The second bout of calf raises with the 30% body weight vest was implemented to increase the muscle activity in the triceps surae (Pincheira et al. 2023), as pilot testing (*n* = 3) indicated a single bout of body-weighted calf raises was insufficient in reducing plantarflexion power. During calf raises, participants were instructed to maintain a straight leg, lift their heel as high as possible, and stay in time to a 60 bpm metronome (30 calf raise repetitions per minute). Finally, a post-fatigue plantarflexion power test was completed with the right leg. The Calf Raise App (version 1.5.1) recorded all calf raise repetitions and total positive work (J) at 60 frames per second within the fatigue protocol, and was also used to extract peak plantarflexion power (W) from the power tests by tracking a 24 mm black sticker place within a 32 mm white sticker positioned below the lateral malleolus, as described elsewhere (Hébert-Losier et al. 2022b). Perceived plantarflexor fatigue and soreness ratings were recording using a 100 mm visual analogue scale (VAS) administered via Qualtrics (Qualtrics, Provo, UT (17.1.7) immediately after the final plantarflexion power test, which was followed by a single six-minute RE test in one of the two shoe conditions. Participants then completed another round of the plantarflexion fatigue protocol, inclusive of pre-and-post plantarflexion power tests and VAS assessment, and another RE test in the opposite shoe condition. Post-fatigue RE shoe conditions were counterbalanced across participants. Sagittal plane running footage and blood lactate concentration samples were also collected from post-fatigue RE tests. Following completions of this second session, participants were given a NZ$30 petrol voucher and went in a draw to receive one of the experimental shoes.

### Data processing

During the final two minutes of each RE test, oxygen consumption, carbon dioxide exhalation, and RER were recorded and averaged. These averages were then used to calculate oxygen consumption (mL/kg/min), energy cost (EC) (W/kg) using the Péronnet and Massicotte (1991) equation, and energetics cost of transport (ECT) (J/kg/m) based on the running speed of each individual.

Pre-fatigue RE measures were averaged within shoe conditions, resulting in one pre- and one post-fatigue RE measure for each shoe. If RER exceeded 1.0 or blood lactate exceeded 4.0 mmol/L, the RE measures from that test were removed. The Calf Raise App generated the number of repetitions and total positive work (J) for the calf raises to volitional exhaustion within the fatigue protocol, and peak concentric power (W) for the baseline, pre-fatigue, and post-fatigue weighted plantarflexion power tests. Spatiotemporal and foot strike angle biomechanical measures were extracted from running footage using Kinovea (2023.1), a method demonstrating good to excellent intra-rater, inter-rater, and test-retest reliability for related gait parameters (Spanos et al. 2023). For each RE test, the first 20 ground contact and toe-off timestamps were recorded and averaged to determine cycle time, ground contact time, and duty factor (ratio of ground contact time to cycle time), while foot strike angles were derived from averaging the first 10 ground contacts. Participants were categorised as rearfoot strikers when their foot strike angles were greater than 8° and non-rearfoot strikers when 8° or less (Altman and Davis 2012). Test-retest relative reliability assessed on 120 paired running trials in our laboratory (i.e., comparing two trials from participants who ran twice at 70% of *v*VO_2peak_ in the same shoes) using this 2D motion capture set-up is excellent based on intraclass correlation coefficients ranging from 0.94 to 0.97 for the extracted measures, with corresponding typical errors for contact time, cycle time, foot-strike angle, and duty factor being 5.7 ms, 10.5 ms, 1.4°, and 0.9%.

### Statistical analysis

R (version 4.4.2) and Microsoft Excel were used to explore the data. Considering the relatively large sample size (Lumley et al. 2002; Prescott 2019), approximate normality of residuals from statistical analyses were verified and confirmed using graphical methods (dot plots, Q-Q plots, histograms, and box plots). Paired *t*-tests were conducted to assess potential shoe condition order effects across all RE measures, plantarflexion power tests, VAS plantarflexor fatigue and soreness scores, and biomechanics data. Multiple two-way repeated measures analysis of variance (ANOVA) were used to examine the main effects of shoe (Control, AFT) and time (pre-fatigue, post-fatigue), and their interaction (shoe x time) on RE measures, plantarflexion power, VAS, and biomechanics measures. Participant ID set the between-subjects error term, with shoe and time as within-subjects factors. Effect sizes for the repeated measures ANOVA were reported as partial eta squared (*η*_*p*_*²*) with 95% confidence intervals, and were defined as *small*, *moderate*, and *large* when reaching 0.01, 0.06, and 0.14, respectively (Cohen 1988).

Pearson correlations were used to explore the relationship between baseline plantarflexion power and the percentage difference in oxygen consumption, EC, and ECT between shoes conditions, for both pre-fatigue and post-fatigue RE measures (six correlations in total). Furthermore, Pearson correlations were used to explore the relationship between the difference in the two post-fatigue plantarflexion power tests (expressed as a percentage of baseline plantarflexion power) and the difference in the RE responses to AFT pre-to-post plantarflexion fatigue. Correlations were considered *trivial*, *small*, *moderate*, and *large* when the *r*-value reached < 0.10, 0.10, 0.30, and 0.50, respectively (Cohen 1992). Statistical significance was set to *P* ≤ 0.05.

## Results

### Participants

Eighty-two runners were recruited for this study. However, eight participants did not complete session two and a further ten participants were removed from analysis due to equipment malfunctioning. Hence, 64 participants (Table 2) were included in analyses. Two participants pre-fatigue RE measures exceeded the RER threshold in both shoe conditions (five tests in total). As a result, one set of pre-fatigue RE measures was removed, and one participant’s RE measures were derived from a single test instead of an average of two. Furthermore, two participants exceeded the blood lactate concentration threshold in at least one shoe condition (three trials total), leading to two sets of post-fatigue RE measures being removed from analyses. During the RE tests at 70% of *v*VO_2peak_, the average speed of participants was 10.8 ± 1.5 km/h (range: 8.4 to 14.7 km/h). Of the 64 participants, 9 categorised themselves as sprinters, 12 middle distance runners, and 43 long distance runners. Furthermore, 33 participants were considered recreational, 27 experienced, and 4 national level runners.

### Experimental results

Baseline plantarflexion fatigue power was 464.2 ± 169.1 W. During the fatigue protocol (Table 2), participants performed an average of 40.3 ± 23.2 bodyweight calf raises and 20.0 ± 7.6 weighted calf raises, generating 2138.9 ± 1084.3 J and 1255.1 ± 464.7 J of work, respectively. Plantarflexion power decreased by 6.5% post-fatigue (428.9 ± 155.2 W) compared to pre-fatigue (459.0 ± 168.0 W), with a *moderate* effect size (*F*_(1, 183)_ = 19.0, *P* < 0.001, *η*_*p*_*²* = 0.09). The plantarflexion fatigue protocol resulted in elevated perceived plantarflexor fatigue (81.1 ± 19.9 mm) and plantarflexor soreness (68.8 ± 27.7 mm) compared to baseline measures (16.8 ± 20.4 and 15.9 ± 21.4 mm, respectively), with *large* effect size differences between times (*F*_(1, 189)_ ≥ 464.9, *P* < 0.001, *η*_*p*_*²* ≥ 0.71).

Paired *t*-tests found no significant shoe order effect on pre-fatigue and post-fatigue oxygen consumption (*P =* 0.971 and 0.516, respectively), EC (*P =* 0.991 and 0.466, respectively), ECT (*P =* 0.954 and 0.373, respectively), plantarflexion power (*P =* 0.621 post-fatigue), ground contact time (*P =* 0.280 and 0.863, respectively), cycle time (*P =* 0.339 and 0.822, respectively), foot strike angle (*P =* 0.429 and 0.614, respectively), and duty factor (*P =* 0.908 and 0.405, respectively) measures. However, plantarflexor fatigue and soreness VAS scores presented a shoe order effect (*P* = 0.004 and 0.008, respectively), with scores increasing after the second plantarflexion fatigue protocol.

**Table 2:** Baseline characteristics and fatigue protocol metrics from participants (*n* = 64)

	All (*n* = 64)			Male (*n* = 32)			Female (*n* = 32)		
	Mean ± SD	Min	Max	Mean ± SD	Min	Max	Mean ± SD	Min	Max
Baseline characteristics									
Age (y)	33.5 ± 15.6	14.6	72.2	38.4 ± 18.2	15.1	72.2	28.7 ± 10.7	14.6	49.2
Mass (kg)	69.5 ± 11.9	46.8	102.7	77.5 ± 8.6	61.9	102.7	61.4 ± 9.1	46.8	88.6
Height (cm)	172.3 ± 7.9	158.0	191.5	178.0 ± 5.9	165.5	191.5	166.6 ± 5.0	158.0	178.0
Milage per week (km)	33.6 ± 24.8	2.0	90.0	36.8 ± 26.2	3.0	90.0	30.5 ± 23.3	2.0	90.0
Years running (y)	8.3 ± 8.3	0.5	45.0	9.9 ± 9.8	0.5	45.0	6.7 ± 6.4	0.5	25.0
5 km personal best (min)	22.9 ± 4.1	15.5	36.0	21.4 ± 3.9	15.5	30.0	24.2 ± 3.9	18.0	36.0
Baseline plantarflexion power (W)	464.2 ± 169.1	191.8	874.8	546.3 ± 174.8	191.8	874.8	384.7 ± 119.9	207.4	738.9
Peak oxygen uptake (mL/kg/min)	49.4 ± 8.3	33.1	72.3	53.4 ± 8.8	36.9	72.3	45.4 ± 5.3	33.1	56.7
*v*VO_2peak_ (km/h)	15.5 ± 2.2	12.0	21.0	16.4 ± 2.4	12.0	21.0	14.6 ± 1.4	12.0	17.0
70% *v*VO_2peak_ (km/h)	10.8 ± 1.5	8.4	14.7	11.4 ± 1.7	8.4	14.7	10.2 ± 1.0	8.4	11.9
Body weight calf raises (count)	40.3 ± 23.2	11	213	40.6 ± 21.6	19	153	40.0 ± 24.8	11	213
Weighted calf raise (count)	20.0 ± 7.6	7	71	21.3 ± 8.7	8	71	18.8 ± 6.0	7	45
Body weight calf raise work (J)	2138.9 ± 1084.3	110.2	8474.2	2327.4 ± 994.0	110.2	6193.0	1953.4 ± 1140.2	876.2	8474.2
Weighted calf raise work (J)	1255.1 ± 464.7	118.5	3214.6	1457.5 ± 480.1	603.4	3214.6	1060.6 ± 354.8	118.5	2062.9

Data are means ± standard deviations with ranges

SD, standard deviation; 70% *v*VO_2peak_, 70% of the velocity at peak oxygen uptake

**Fig. 3:**
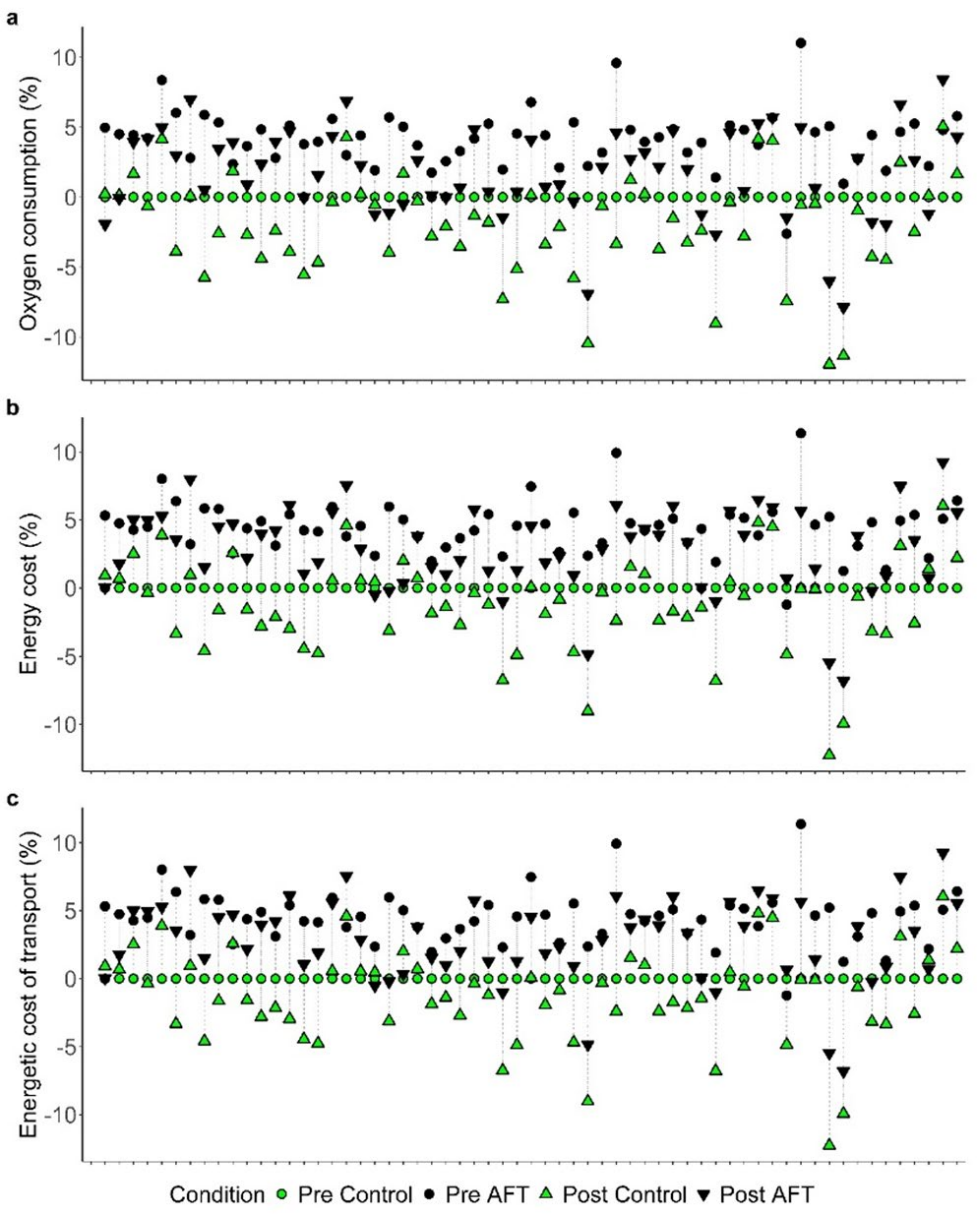
Individual RE responses for (**a**) oxygen consumption, (**b**) energy cost, and (**c**) energetic cost of transport. All data are presented as a relative percentage difference from each individual’s pre-fatigue Control RE measure. Grey dotted lines connect data from the same participant: green circles (pre-fatigue Control), black circles (pre-fatigue AFT), green triangles (post-fatigue Control), and black triangles (post-fatigue AFT). Negative values represent worse RE measures than the pre-fatigue Control condition. *Abbreviations*: AFT, Salomon Phantasm S/Lab 2; Control, Salomon Aero Glide 2; RE, running economy

Descriptive statistics from session two are displayed in Table 3, and repeated measures ANOVA results in Table 4. No interaction effects were found across all RE, plantarflexion, VAS, and biomechanics measures (*P* ≥ 0.383). *Large* effect size improvements in RE measures (*F*_(1, 180)_ ≥ 210.3, *P* < 0.001, *η*_*p*_*²* ≥ 0.54) were observed for oxygen consumption, EC, and ECT in AFT (35.3 ± 5.0 mL/kg/min, 12.5 ± 1.8 W/kg, and 4.18 ± 0.40 J/kg/m, respectively) compared to the Control (36.8 ± 5.2 mL/kg/min, 13.1 ± 1.9 W/kg, and 4.37 ± 0.40 J/kg/m, respectively) shoe, corresponding to + 4.0 to + 4.3% (range: +0.2 to + 9.1%) improvements. In contrast, plantarflexion fatigue significantly impaired RE measures; with oxygen consumption, EC, and ECT superior pre-fatigue (35.7 ± 5.1 mL/kg/min, 12.7 ± 1.8 W/kg, and 4.25 ± 0.40 J/kg/m, respectively) compared to post-fatigue (36.4 ± 5.2 mL/kg/min, 12.8 ± 1.9 W/kg, and 4.30 ± 0.42 J/kg/m, respectively), corresponding to -1.5 to -2.3% (range: -11.8% to + 2.4%) detriments to RE measures. All RE measure differences between pre- and post-fatigue time had *large* effect sizes (*F*_(1, 180)_ ≥ 29.0, *P* < 0.001, *η*_*p*_*²* ≥ 0.14). Figure 3 illustrates individual RE responses to shoe condition and plantarflexion fatigue states.

Baseline plantarflexion power did not corelate with pre-fatigue or post-fatigue oxygen consumption (*r* ≤ 0.08, *P* ≥ 0.560), EC (*r* ≤ 0.06, *P* ≥ 0.663), ECT (*r* ≤ 0.06, *P* ≥ 0.663) responses to AFT. The change in post-fatigue plantarflexion power did not correlate with RE response to AFT (*r* ≤ 0.03, *P* ≥ 0.820).

Significantly quicker ground contact times, longer cycle times, smaller foot strike angles, and lower duty factors were observed in the AFT (270.1 ± 30.4 ms, 726.1 ± 43.0 ms, 5.0 ± 7.5°, and 37.3 ± 4.4%, respectively) compared to the Control (273.4 ± 30.7 ms, 720.4 ± 42.9 ms, 6.5 ± 7.1°, and 38.0 ± 4.5%, respectively) shoe, all with *large* effect size differences between shoes (*F*_(1, 174)_ ≥ 28.5, *P* < 0.001, *η*_*p*_*²* ≥ 0.14). Quicker cycle times and larger duty factors were seen post-fatigue (720.0 ± 42.9 ms and 37.8 ± 4.5%, respectively) compared to pre-fatigue (726.5 ms and 37.5 ± 4.5%, respectively), with corresponding *large* (*F*_(1, 174)_ = 41.4, *P* < 0.001, *η*_*p*_*²* = 0.19) and *small* (*F*_(1, 174)_ = 6.7, *P* = 0.010, *η*_*p*_*²* = 0.04) effect size differences. No significant different in all other biomechanical measures were noted between shoe conditions and pre-to-post fatigue (Table 4). It is noteworthy that significant differences in ground contact (i.e., 3.3 ms) and cycle (i.e., 5.7 to 6.5 ms) times were less than or close to the video resolution errors (i.e., 4.2 ms).

**Table 3:** Descriptive data from participants

	Control						AFT					
	Pre-fatigue			Post-fatigue			Pre-fatigue			Post-fatigue		
	*Mean* ± *SD*	*Min*	*Max*	*Mean* ± *SD*	*Min*	*Max*	*Mean* ± *SD*	*Min*	*Max*	*Mean* ± *SD*	*Min*	*Max*
Oxygen consumption (mL/kg/min) (*n*, 60)	36.4 ± 5.1	23.8	47.3	37.2 ± 5.4	24.6	48.9	34.9 ± 5.0	21.5	45.3	35.8 ± 5.1	22.7	46.7
Energetic cost (W/kg) (*n*, 60)	13.0 ± 1.8	8.5	16.9	13.1 ± 1.9	8.7	17.2	12.4 ± 1.8	7.7	16.2	12.6 ± 1.8	8.0	16.5
Energetic cost of transport (J/kg/m) (*n*, 60)	4.3 ± 0.4	3.5	5.4	4.4 ± 0.4	3.2	5.2	4.2 ± 0.4	3.3	5.5	4.2 ± 0.4	3.3	5.3
Plantarflexion power (W) (*n*, 62)	460.6 ± 174.0	199.9	919.0	425.7 ± 158.9	159.2	874.4	457.4 ± 163.3	200.7	935.7	432.2 ± 152.5	204.6	943.8
VAS plantarflexor fatigue (mm) (*n*, 64)	16.8 ± 20.5^†^	0^†^	84^†^	80.0 ± 19.7	13	100	16.8 ± 20.5^†^	0^†^	84^†^	82.2 ± 20.2	2	100
VAS plantarflexor soreness (mm) (*n*, 64)	15.9 ± 21.5^†^	0^†^	89^†^	66.6 ± 28.1	3	100	15.9 ± 21.5^†^	0^†^	89^†^	70.9 ± 27.3	7	100
Ground contact time (ms) (*n*, 59)	273.7 ± 31.0	212.3	360.0	273.1 ± 30.9	208.3	350.3	270.5 ± 30.8	213.5	351.2	269.6 ± 30.4	202.2	350.1
Cycle time (ms) (*n*, 59)	723.5 ± 43.3	636.0	820.8	717.3 ± 42.9	629.2	814.7	729.6 ± 42.8	636.6	825.4	722.7 ± 43.8	637.1	811.0
Foot strike angle (°) (*n*, 59)^‡^	6.6 ± 7.5	-11.7	19.2	6.5 ± 6.9	-8.2	16.0	5.0 ± 7.8	-13.0	16.3	5.0 ± 7.2	-11.2	15.8
Foot strike pattern (*n*, 59)^§^	24 : 35	–	–	24 : 35	–	–	24 : 35	–	–	24 : 35	–	–
Duty factor (%) (*n*, 59)	37.9 ± 4.5	28.8	52.0	38.2 ± 4.6	28.4	52.1	37.2 ± 4.5	28.9	52.1	37.4 ± 4.5	27.3	50.2

Data are means ± standard deviations (*SD*). VAS scores range from 0 to 100, where higher values indicate more plantarflexor soreness or fatigue

AFT, Salomon Phantasm S/Lab 2; Control, Salomon Aero Glide 2; VAS, visual analogue scale

^†^Pre-fatigue data for VAS collected before running economy tests

^‡^More positive numbers represent more pronounced heel striking

^§^Foot strike patterns are presented as the number of rearfoot strikers (foot strike angle > 8°) and non-rearfoot strikers (foot strike angle ≤ 8°)

**Table 4:** Repeated measures analysis of variance results from participants data (*n*, 64)

	Raw difference	F-value	*P*-value	η_*p*_² [95% CI]
Oxygen consumption (mL/kg/min) (*n*, 61)				
Shoe	1.4 ± 0.6	211.6	< 0.001*	0.54 [0.45, 0.62]
Time	-0.8 ± 1.1	70.5	< 0.001*	0.28 [0.18. 0.38]
Shoe x Time	–	0.6	0.443	0.00 [0.00, 0.04]
Energetic cost (W/kg) (*n*, 61)				
Shoe	0.6 ± 0.2	265.1	< 0.001*	0.60 [0.51, 0.66]
Time	-0.2 ± 0.4	30.6	< 0.001*	0.15 [0.06, 0.24]
Shoe x Time	–	0.4	0.387	0.00 [0.00, 0.04]
Energetic cost of transport (J/kg/m) (*n*, 61)				
Shoe	0.2 ± 0.1	252.9	< 0.001*	0.58 [0.50, 0.65]
Time	-0.1 ± 0.1	29.2	< 0.001*	0.14 [0.06, 0.23]
Shoe x Time	–	0.4	0.513	0.00 [0.00, 0.04]
Plantarflexion power (W) (*n*, 62)				
Shoe	-0.6 ± 52.1	0.0	0.929	0.00 [0.00, 0.01]
Time	-30.0 ± 61.5	19.0	< 0.001*	0.09 [0.03, 0.18]
Shoe x Time	–	0.4	0.503	0.00 [0.00, 0.04]
VAS plantarflexor fatigue (mm) (*n*, 64) ^†^				
Shoe	-1.1 ± 10.6	0.3	0.575	0.00 [0.00, 0.03]
Time	-64.3 ± 24.9	1172.9	< 0.001*	0.86 [0.83, 0.89]
Shoe x Time	–	0.3	0.575	0.00 [0.00, 0.03]
VAS plantarflexor soreness (mm) (*n*, 64) ^†^				
Shoe	-2.1 ± 16.3	0.8	0.383	0.00 [0.00, 0.04]
Time	-52.9 ± 32.0	464.9	< 0.001*	0.71 [0.65, 0.76]
Shoe x Time	–	0.5	0.383	0.00 [0.00, 0.04]
Ground contact time (ms) (*n*, 59)				
Shoe condition	3.3 ± 4.5	28.5	< 0.001*	0.14 [0.06, 0.24]
Time	0.7 ± 6.1	1.3	0.251	0.00 [0.00, 0.05]
Shoe x Time	–	0.1	0.818	0.00 [0.00, 0.02]
Cycle time (ms) (*n*, 59)				
Shoe	-5.7 ± 9.5	32.1	< 0.001*	0.16 [0.07, 0.25]
Time	6.5 ± 8.2	41.4	< 0.001*	0.19 [0.10, 0.29]
Shoe x Time	–	0.1	0.750	0.00 [0.00, 0.03]
Foot strike angle (°) (*n*, 59) ^‡^				
Shoe	1.5 ± 2.0	62.5	< 0.001*	0.26 [0.16, 0.37]
Time	0.1 ± 1.5	0.1	0.736	0.00 [0.00, 0.03]
Shoe x Time	–	0.0	0.927	0.00 [0.00, 0.01]
Duty factor (%) (*n*, 59)				
Shoe condition	0.76 ± 0.70	65.9	< 0.001*	0.27 [0.17, 0.38]
Time	-0.24 ± 0.90	6.7	0.010*	0.04 [0.00, 0.11]
Shoe x Time	–	0.0	0.958	0.00 [0.00, 0.00]

Positive values indicate larger Control (shoe) or pre-fatigue (time) values. VAS scores range from 0 to 100, where higher values indicate more plantarflexor soreness or fatigue. Raw differences reported as means ± standard deviations (*SD*)

Time, pre-fatigue and post-fatigue; VAS, visual analogue scale; *η*_*p*_*²*, partial eta squared

^†^Pre-fatigue data are collected before running economy tests and are the same in both shoe conditions

^‡^Positive values indicate greater heel striking in the Control shoe, negative values indicate greater heel striking in the AFT shoe

*Significant effect (*P* < 0.050)

## Discussion

We investigated the influence of plantarflexion fatigue on AFT shoe response. Running in AFT versus Control shoes resulted in an average 4.0 to 4.3% improvement in RE measures (individual responses ranged from 0.5 to 9.1%). This overall improvement in RE measures with AFT shoes aligns with previous research using other AFT shoe models (Hébert-Losier et al. 2022a; Hoogkamer et al. 2018; Hunter et al. 2019; Joubert et al. 2024; Knopp et al. 2023). Opposing our hypothesis, there was no significant difference in RE response to AFT shoes before and after plantarflexion fatigue, despite the overall average 1.6 to 2.4% impairment in RE with plantarflexion fatigue. Previous research using the same metabolic analyser found the smallest worthwhile change in oxygen consumption, EC, and ECT to be 1.6, 1.7, and 1.4% respectively (Hébert-Losier et al. 2022a). Therefore, changes in RE measures are not only statistically significant, but also practically meaningful, highlighting the importance of the triceps surae muscle tendon unit on RE measures and potentially running performance. Declines in RE and plantarflexion function have been identified in previous research, with RE declining by 13.6% after a marathon (Kyröläinen et al. 2000) and by 3.0 to 6.6% after 60 min of steady running (Sproule 1998) and plantarflexion maximal voluntary isometric contraction strength declining by 29.8 N following two hours of running (Avela et al. 1999) and 25.9 Nm after a marathon (Saldanha et al. 2008). Fatigue aetiology is task dependent (Hunter 2018) and while these referenced studies reveal running-induced plantarflexion fatigue, we instead show that inducing plantarflexion fatigue using repeated calf raises impacts RE.

We also explored the relationship between plantarflexion power and RE response to AFT shoes, identifying no significant correlation with RE responses and plantarflexion power or fatigue. These findings oppose speculations from authors that runners need a certain level of plantarflexion strength to fully benefit from shoes with an implemented stiff element (Madden et al. 2016; Ortega et al. 2021). Altogether, these findings suggest RE response to AFT shoes may not change throughout a long-distance race with the accumulation of plantarflexion fatigue from prolonged running, as shown in recent studies comparing carbon-plated shoes to non-carbon-plated shoes (Madsen et al. 2025; Perrin et al. 2025).

Evidence regarding the effects of running in AFT on spatiotemporal parameters at a fixed running speed seems inconsistent, specifically with regards to increased longitudinal bending stiffness (Ortega et al. 2021). Nonetheless, other studies have also found faster ground contact times (Schwalm et al. 2025), longer cycle times (Day and Hahn 2020; Hoogkamer et al. 2018, 2019; Schwalm et al. 2025; Werkhausen et al. 2024), smaller foot strike angles (Kim and Ahn 2025), and potentially lower duty factors based on data reported (Healey and Hoogkamer 2022) when wearing AFT. However, most studies report longer ground contact times when wearing shoes with increased longitudinal bending stiffness or wearing AFT shoes (Beck et al. 2020; Cigoja et al. 2019; Day and Hahn 2020; Healey and Hoogkamer 2022; Hoogkamer et al. 2018; Oh and Park 2017; Werkhausen et al. 2024). Inconsistent findings are also reported with regards to plantarflexion fatigue on spatiotemporal measures, with studies reporting no significant changes to cycle time (Alfuth and Rosenbaum 2011) and longer cycle times with running induced plantarflexion fatigue (García-Pérez et al. 2013; Gerlach et al. 2005). However, our studies identified increased cycle time with plantarflexion fatigue. Although we identified significant differences between shoes and plantarflexion fatigue in spatiotemporal and kinematic parameters, some caution is warranted when interpreting these results. The video-based biomechanical measures were below or close to the inter-trial typical errors associated with these measures in our laboratory, and hence, are unlikely to be clinically meaningful. In addition, the differences observed were within or close to the limits of video resolution (4.2 ms).

The interindividual variation we observed in the RE response of runners to AFT shoes (0.5 to 9.1%) is within ranges of variations reported elsewhere. For instance, the RE response to AFT shoes was reported to range from − 9.6 to + 9.7% in recreational runners (Hébert-Losier et al. 2022a), -11.3 to + 11.4% in elite Kenyan runners (Knopp et al. 2023), -1.1 to + 9.7% in amateur European runners (Knopp et al. 2023), and 0.0 to + 6.4% (Hunter et al. 2019) and + 1.59 to + 6.26% (Hoogkamer et al. 2018) in runners who have ran 10 km under 32 min. Differences in study design likely contribute to these differing ranges in RE response to AFT shoes. The average RE response for this study includes three RE tests in each examined shoe, potentially leading to lesser variability in RE response, as those studies with larger variability only examine RE measures from a single test per shoe (Hébert-Losier et al. 2022a; Knopp et al. 2023). Although one test is deemed sufficiently reliable for RE testing, it has been recommended to use at least two RE tests per shoe to limit variability in RE measures (Barrons et al. 2024; Oehlert et al. 2025). Furthermore, this study completed all RE tests for each participant in a single session, whereas other studies used multiple days to complete all RE tests (Hébert-Losier et al. 2022a). Although between-day RE measures are deemed reliable within 2.4% (Saunders et al. 2004), single-day assessments are preferred to reduce intra-individual variability (Barrons et al. 2024). Additionally, the runners who volunteered to participate in our study may simply not have encompassed the range of potential responses to AFT shoes by chance, with no negative responders taking part.

Alongside other methodological variations, shoes acting as the comparator (e.g., Control shoes) differ considerably between studies. Specifically, studies reporting greater RE variations with AFT response used traditional lightweight racing shoes (154 to 197 g) as a comparator (Hébert-Losier et al. 2022a; Knopp et al. 2023), with larger differences in the minimalist index of shoes (i.e., 40% reported in Hébert-Losier et al. (2022a). On this validated minimalist index scale, more minimalist shoes have less technologies and a lower mass, torsional and longitudinal bending stiffness, heel height, and heel-to-toe drop than more maximalist shoes (Esculier et al. 2015). In contrast, studies reporting lesser RE variations with AFT response used leading non-AFT marathon racing shoes at the time (Hoogkamer et al. 2018; Hunter et al. 2019). In the current study, the Control shoe was selected to reflect the AFT shoe design, with an 8% in minimalist index score. The Control shoe was ~ 35 g heavier, which would not meaningfully influence RE (equating to an ~ 0.2 to 0.4% change in RE measures) (Franz et al. 2012). Moreover, footwear mass within 30 g is negligibly correlated to metabolic cost (*r*^*2*^ = 0.012) (Joubert and Jones 2022). The Control shoes also had an 8% lower minimalist index than the AFT shoe. A 70% minimalist index score or more represents a more minimal shoe (Fuller et al. 2017) that could mimic barefoot and alter running biomechanics (Squadrone et al. 2015) compared to more traditionally constructed shoes, i.e., around 20% minimalist index. Therefore, the only meaningful difference between shoe conditions were the presence or absence of AFT. Therefore, comparing variability in AFT responses across studies with different shoe models may not be appropriate, especially as it is poorly understood how AFT components interact to provide their reported benefits (Ghanbari et al. 2025; Healey and Hoogkamer 2022; Hébert-Losier and Pamment 2023).

To assess plantarflexion function, maximal voluntary isometric contractions are commonly used (Avela et al. 1999; Finni et al. 2003; Murray et al. 2019; Petersen et al. 2007; Play et al. 2024; Saldanha et al. 2008). However, this study used weighted plantarflexion power as a more dynamic assessment and running specific test of plantarflexion function (Komi 2000). Furthermore, plantarflexion power was more sensitive in detecting plantarflexion fatigue during pilot testing. In agreement, Leabeater et al. (2024) could not identify significant changes in maximal voluntary isometric contractions following repetitive calf raises, with James et al. (2024) suggesting isometric and dynamic measures lack in agreement and may represent two separate neuromuscular domains. Altogether, plantarflexion power appears to be more appropriate in assessing plantarflexion function in a running context.

Our plantarflexion protocol resulted in 6.5% decline in plantarflexion power. However, evidence suggests prolonged running may induce greater declines (9 to 29%) in maximal voluntary isometric contraction testing (Avela et al. 1999; Finni et al. 2003; Murray et al. 2019; Perrin et al. 2025; Petersen et al. 2007; Play et al. 2024; Saldanha et al. 2008). Partial recovery of the plantarflexors may have affected the post-fatigue RE tests, considering partial recovery from repetitive knee extensions are seen within eight minutes (Husmann et al. 2018). Additionally, muscle fibre typology might also affect the rate of muscle recovery (Lievens et al. 2020), which we were unable to assess in our participants. With more pronounced isolated plantarflexion or running-induced plantarflexion fatigue, altered RE response to AFT may have surfaced, although recent findings indicate sustained RE benefits of increased longitudinal bending stiffness of AFT shoes following a half-marathon compared to standard shoes (Perrin et al. 2025). Nonetheless, our plantarflexion fatigue protocol successfully induced fatigue, indicated by *moderate* effect size reduction in plantarflexion power and *large* effect size increase in perceived plantarflexor fatigue and soreness from pre-to-post fatigue. Although VAS measures presented an order effect, this effect is minimised due to the counterbalanced shoe order of testing.

Future studies should investigate RE response to AFT following running-induced plantarflexion fatigue, rather than isolated plantarflexion fatigue as conducted here, as the relevance of isolated plantarflexion fatigue protocols to running-specific plantarflexion fatigue remains unclear. These future findings may provide further evidence towards how runners response to AFT near the end of a long-distance run.

A strength of this study is the relatively large sample size of 64 participants, notability greater than other research involving AFT (typically 9 to 18 participants) (Hébert-Losier et al. 2022a; Hoogkamer et al. 2018; Hunter et al. 2019; Joubert et al. 2024; Knopp et al. 2023) and plantarflexion fatigue (typically 7 to 24 participants) (Avela et al. 1999; Finni et al. 2003; Murray et al. 2019; Petersen et al. 2007; Play et al. 2024; Saldanha et al. 2008). Furthermore, the even distribution of sex helps address the gap in female representation in sport and exercise research (Cowley et al. 2021; Martínez-Rosales et al. 2021) and AFT research (Mason et al. 2024). However, limitations exist. Although the fatigue protocol successfully induced plantarflexion fatigue and participants all reached comparable volitional fatigue states, considerable variation in calf raise repetitions in our sample was noted, with runners who performed more repetitions possibly experiencing greater neuromuscular fatigue (Varela-Olalla et al. 2024). Despite no significant shoe order effect on physiological and biomechanical measures, participants perceived greater plantarflexor fatigue and soreness after the second plantarflexion fatigue bout, with the residual fatigue and soreness potentially affecting results. Age and sex differences were not investigated within this study, as these factors were beyond the scope of this project. Age typically impairs triceps surae muscle unit function (Karamanidis and Arampatzis 2005; Stenroth et al. 2012). Additionally, previous research found females responded better to AFT than males (Matties and Rowley 2025). Finally, we only collected spatiotemporal biomechanic measures from 2D video footage. Further research using electromyography, 3D motion capture, and force data may provide more valid and reliable insights insight to how running mechanics interact with AFT responses and plantarflexion fatigue.

## Conclusion

Consistent with previous research, running in AFT improved RE by 4.0 to 4.3% on average compared to a Control shoe in a heterogeneous cohort of runners. In contrast, plantarflexion fatigue impaired RE by 1.5 to 2.3%. However, contrary to our hypothesis, plantarflexion fatigue did not significantly influence the RE response to AFT and plantarflexion power was not linked to this response, providing preliminary evidence against the speculation that plantarflexion strength explains some of the variability in AFT response. While RE may decline during prolonged running as plantarflexion fatigue accumulates, these findings suggest that the benefit of AFT remains, though would require confirmation in future studies.

## Data Availability

The datasets generated during and/or analysed during the current study are available from the corresponding author on reasonable request.

## Abbreviations

AFT: Advanced footwear technology
ANOVA: Analysis of variance
EC: Energy cost
ECT: Energetic cost of transport
MVIC: Maximal voluntary isometric contractions
RE: Running economy
RER: Respiratory exchange ratio
SD: Standard deviation
VAS: Visual analogue scale
vVO_2peak_: Velocity at peak oxygen uptake
η_p_²: Partial eta squared
